# Endovascular versus Non-Interventional Therapy for Cervicocranial Artery Dissection in East Asian and Non-East Asian Patients: a Systematic Review and Meta-analysis

**DOI:** 10.1038/srep10474

**Published:** 2015-05-20

**Authors:** Rongzhong Huang, Lingchuan Niu, Ying Wang, Gongwei Jia, Lang Jia, Yule Wang, Wei Jiang, Yang Sun, Lehua Yu

**Affiliations:** 1 Department of Rehabilitation Medicine, the Second Affiliated Hospital of Chongqing Medical University, Chongqing, China; 2 Institute of Ultrasound Imaging, the Second Affiliated Hospital of Chongqing Medical University, Chongqing, China

## Abstract

Endovascular methods have been increasingly applied in treating cervicocranial artery dissection (CCAD). Anti-thrombotic therapy, which is used in non-interventional care of CCAD patients, has differential effects in East Asian patients. Therefore, we aimed to compare the clinical outcomes of endovascular versus non-interventional therapy for CCAD in East Asians and non-East Asians. A search was performed for studies comparing endovascular and non-interventional approaches to CCAD patients. Rates of recovery, disability, and mortality were used to assess these approaches in East Asian and non-East Asian patients. Subgroup analyses were conducted for CCAD patients with ruptured dissections. Eleven East Asian studies and five non-East Asian studies were included. The subgroup analyses for CCAD patients with ruptured dissections on mortality (East Asian odds ratio [OR] [95% confidence interval [CI]]: 0.24 [0.08-0.71], *P* = 0.01; I^2^ = 34%) and good recovery (East Asian OR [95% CI]: 3.79 [1.14-12.60], *P* = 0.03; I^2^ = 54%) revealed that endovascular therapy is significantly superior to non-interventional therapy for East Asians. No differences in treatment effect upon mortality, disability, or good recovery outcomes were found for the CCAD populations-at-large nor for non-East Asian CCAD patients with ruptured dissections. Endovascular therapy appears to be superior to non-interventional therapy for East Asian CCAD patients with ruptured dissections.

Cervicocranial artery dissection (CCAD) involves a tearing of a cervical or cerebral artery that leads to a mural hematoma within the arterial wall and typically presents with unilateral headache, oculosympathetic palsy, amaurosis fugax, and symptoms of focal brain ischemia^1^. CCAD has a relatively low annual prevalence of 2.6-5 per 100,000 but accounts for 25% of strokes in patients aged under 45 years old^2^. Etiologically, CCADs can arise spontaneously or from traumatic neck injury, underlying aneurysms, or as a complication following endovascular interventions such as atraumatic subarachnoid hemorrhage (SAH) patients undergoing endovascular coiling repair^3^.

In terms of current treatment approaches for CCAD, endovascular methods (e.g., intra-arterial thrombolysis, angioplasty, and stent placement) have been increasingly applied in treating and preventing the thromboembolic complications of CCAD^4^. However, it has not been clear that endovascularly-treated CCAD patients would have fared worse outcomes if they had continued conservative therapy (i.e., non-interventional care involving anti-thrombotic therapy and/or other drugs)^4^. To address this question, a recent meta-analysis by Chen *et al.* demonstrated that patients who received endovascular treatment experienced a lower mortality rate than those patients who received non-interventional care, especially in patients with ruptured CCADs or dissecting aneurysms^5^.

Although Chen *et al.*’s findings support the use of endovascular treatment over non-interventional care (such as anti-thrombosis) in CCAD patients, they do not address the effect of ethnicity upon patient outcomes. This question is clinically relevant, as anti-thrombotic therapy has been conclusively shown to have differential effects in East Asian patients^6^,^7^. Therefore, the aim of this systematic review and meta-analysis will be to compare the clinical outcomes of endovascular versus non-interventional therapy for CCAD in East Asian and non-East Asian populations.

## Materials and Methods

### Literature Search

This study was conducted according to the PRISMA guidelines^8^. A literature search was performed on Medline, Embase, and the Cochrane Library databases through November 2014. The following search terms were used: (“cervicocranial artery dissection” OR “cerebral artery dissection” OR “internal carotid artery dissection” OR “vertebrobasilar artery dissection” OR “vertebral artery dissection” OR “basilar artery dissection” OR “anterior cerebral artery dissection” OR “middle cerebral artery dissection” OR “posterior artery dissection”) AND (“treatment” OR “therapy”). Reference lists from the eligible studies were also searched for additional records.

### Selection Criteria

The following studies were included: (i) patients diagnosed with CCAD by one of the following standard imaging modalities (i.e., computed tomography (CT) angiography, magnetic resonance (MR) angiography, arterial angiography, MR imaging, or duplex scanning); (ii) comparing 10 or more CCAD patients that received either endovascular treatment (i.e., any arterial reconstructive/deconstructive procedure such as stenting, proximal arterial occlusion, or arterial thrombolysis) or non-interventional treatment (i.e., any non-surgical or non-endovascular treatment such as antithrombotic therapy, blood pressure control, palliative care, or no treatment); and (iii) reporting at least one outcome of interest (see “Outcomes” subsection below).

The following studies were excluded: (i) CCAD patients treated through several methods; (ii) CCAD patients treated with surgery; (iii) conference abstracts/summaries, case reports/series, reviews, and commentaries/editorials; and (iv) non-English articles.

### Risk of Bias Assessment

Risk of bias for each study was independently assessed by two co-authors using a modified Newcastle Ottawa Scale (NOS) for non-randomized studies^9^.

### Data Extraction

Data extraction was independently completed by two authors, and disagreements were resolved by consensus. The following data was extracted from each study: author, publication year, country, study design, study size, study duration, patient characteristics, treatment modality, follow-up duration, and outcomes.

### Outcomes

Rates of recovery, disability, and mortality were used to assess endovascular treatment versus non-interventional treatment in East Asian and non-East Asian patient populations. Functional outcomes were assessed by the Glasgow Outcome Scale (GOS), modified Rankin Scale (mRS), Karnofsky Performance Score (KPS), or other criteria^10^. Specifically, according to Chen *et al.*’s criteria^5^, overall outcomes were defined as follows: ‘good recovery’ was defined as a GOS score of 5, mRS score of 0-1, or KPS score of 80-100; ‘disability’ was defined as a GOS score of 2-4, mRS score of 2-5, or KPS score of 10-70; and ‘mortality’ was defined as all-cause mortality. If none of the foregoing scoring methods were applied, patients with improved outcomes or those with permanent neurologic deficits were conservatively categorized under the ‘disability’ outcome. Patients deemed ‘excellent’ were categorized under the ‘good recovery’ outcome.

### Statistical Analysis

Statistical analyses were performed using RevMan 5.0.24 (Cochrane Collaboration, Denmark) with *P*-values of less than 0.05 deemed statistically significant. Meta-analysis was performed to compare outcomes of patients treated endovascular therapy versus non-interventional therapy. Results were reported as odds ratio (OR) and associated 95% confidence interval (CIs). Heterogeneity was measured using the Q-test and the I^2^ statistic (with values of 25%, 50%, and 75% representing low, medium, and high heterogeneity)^11^. The random-effects model was used if there was high heterogeneity between studies; otherwise, the fixed-effects model was used^12^. For comparisons with medium-to-high heterogeneity (I^2^ > 50%), sensitivity analysis was performed to investigate possible sources of heterogeneity.

Then, the pooled outcomes were compared between ‘East Asian’ and ‘non-East Asian’ studies (with ‘East Asian’ conservatively defined as Chinese, Japanese, and Korean^13^) in order to analyze the effects of East Asian ethnicity upon the efficacy of endovascular therapy vis-a-vis non-interventional therapy. Sensitivity analysis was performed by iteratively removing one study at a time to confirm that our findings were not driven by any single study. Visual inspection of funnel plots followed by Egger’s and Begg’s testing were used to assess publication bias^14^.

## Results

**Figure 1:**
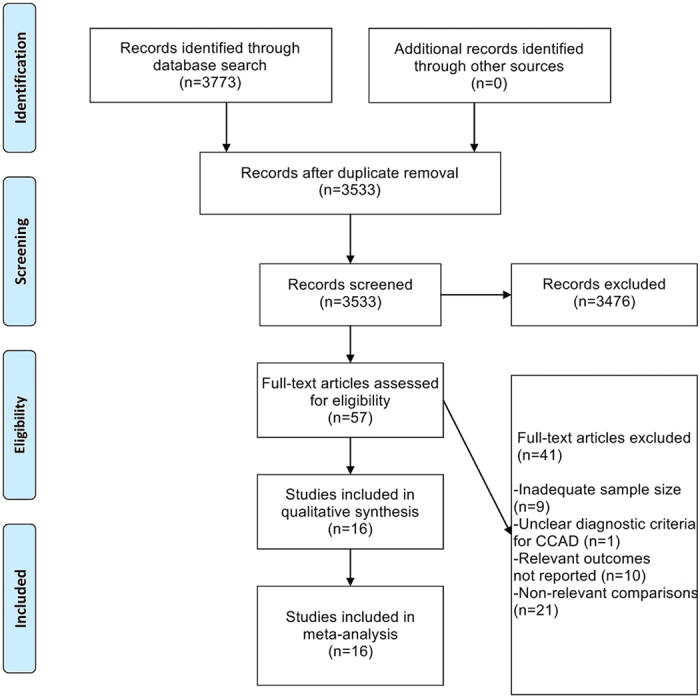
Flowchart of Study Selection .

The initial literature search produced 3773 records (Fig. 1). After elimination of duplicates and non-relevant records, 57 full-text articles were reviewed. After application of all inclusion and exclusion criteria, 16 studies (i.e., eleven East Asian studies^15^,^16^,^17^,^18^,^19^,^20^,^21^,^22^,^23^,^24^,^25^ and five non-East Asian studies^26^,^27^,^28^,^29^,^30^) were finally included in this meta-analysis (Table 1). The quality assessment for these included studies is detailed in Table 2.

**Figure 2:**
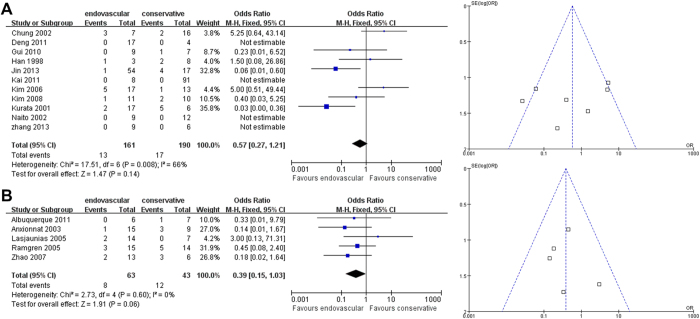
**Analysis of Overall Mortality Outcomes.** Forest plots and funnel plots of (**A**) East Asian and (**B**) non-East Asian studies.

First, the pooled outcomes for mortality for endovascular therapy versus non-interventional therapy were separately compared in East Asian and non-East Asian studies. Both East Asians and non-East Asians showed no differences in treatment effect between endovascular therapy versus non-interventional therapy on mortality outcomes (East Asian OR [95% CI]: 0.57 [0.27-1.21], *P* = 0.14, Fig. 2A; non-East Asian OR [95% CI]: 0.39 [0.15-1.03], *P* = 0.06; Fig. 2B). For the East Asian comparison, there was significant heterogeneity (I^2^ = 66%, Fig. 2A). Sensitivity analysis to investigate possible sources of heterogeneity in the included studies indicated that no single study was an important source of heterogeneity; that is, exclusion of no individual study from the overall meta-analysis significantly changed the *p*-value of heterogeneity. For the East Asian comparison, Begg’s test (*P* = 1.000) and Egger’s test (*P* = 0.771) revealed no significant publication bias. For the non-East Asian mortality analysis (Fig. 2B), Begg’s test (*P* = 0.296) and Egger’s test (*P* = 0.034) revealed that publication bias may exist.

**Figure 3:**
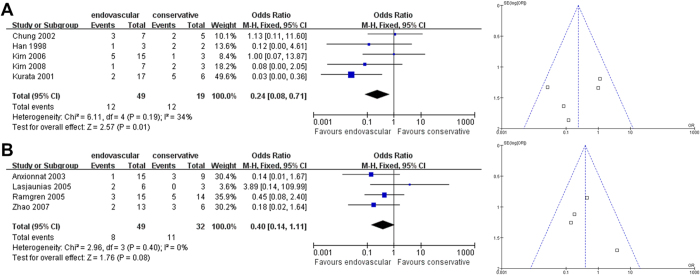
**Analysis of Subgroup Mortality Outcomes for Patients with Ruptured Dissections.** Forest plots and funnel plots of (**A**) East Asian and (**B**) non-East Asian studies.

However, the subgroup mortality analysis for CCAD patients with ruptured dissections revealed that endovascular therapy is significantly superior to non-interventional therapy for East Asians (East Asian OR [95% CI]: 0.24 [0.08-0.71], *P* = 0.01; Fig. 3A) with low-to-medium heterogeneity between the included studies (I^2^ = 34%). No differences in treatment effect on mortality outcomes were observed between the two approaches for non-East Asian CCAD patients with ruptured dissections (non-East Asian OR [95% CI]: 0.40 [0.11-1.11], *P* = 0.08; Fig. 3B). For the East Asian comparison, Begg’s test (*P* = 1.000) and Egger’s test (*P* = 0.765) revealed no significant publication bias. For the non-East Asian mortality subgroup analysis for ruptured dissections (Fig. 3B), Begg’s test (*P* = 0.296) and Egger’s test (*P* = 0.034) revealed that publication bias may exist.

**Figure 4:**
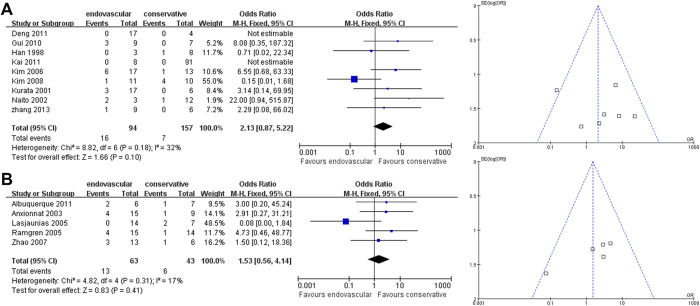
**Analysis of Overall Disability Outcomes.** Forest plots and funnel plots of (**A**) East Asian and (**B**) non-East Asian studies.

Second, the pooled outcomes for disability for endovascular therapy versus non-interventional therapy were separately compared in East Asian and non-East Asian studies. Both East Asians and non-East Asians showed no differences in treatment effect between endovascular therapy versus non-interventional therapy on disability outcomes (East Asian OR [95% CI]: 2.13 [0.87-5.22], *P* = 0.10, Fig. 4A; non-East Asian OR [95% CI]: 1.53 [0.56-4.14], *P* = 0.41, Fig. 4B). For the non-East Asian comparison (Fig. 4B), sensitivity analysis revealed that the summary effect estimates and 95% CI significantly changed (*p* < 0.05), indicating that this particular finding was not particular robust. For the East Asian comparison, Begg’s test (*P* = 1.000) and Egger’s test (*P* = 0.787) revealed no significant publication bias. For the non-East Asian comparison, Begg’s test (*P* = 0.308) and Egger’s test (*P* = 0.542) revealed no significant publication bias.

**Figure 5:**
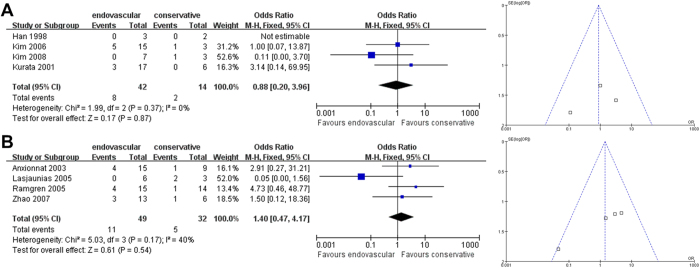
**Analysis of Subgroup Disability Outcomes for Patients with Ruptured Dissections.** Forest plots and funnel plots of (**A**) East Asian and (**B**) non-East Asian studies.

The subgroup disability analysis for CCAD patients with ruptured dissections also revealed no differences in treatment effect between endovascular therapy versus non-interventional therapy on disability outcomes for both East Asians and non-East Asians (East Asian OR [95% CI]: 0.88 [0.20-3.96], *P* = 0.87, Fig. 5A; non-East Asian OR [95% CI]: 1.40 [0.47-4.17], *P* = 0.54, Fig. 5B). For the East Asian comparison, Begg’s and Egger’s test could not be performed due to insufficient data. For the non-East Asian comparison, Begg’s test (*P* = 0.296) and Egger’s test (*P* = 0.166) revealed no significant publication bias.

**Figure 6:**
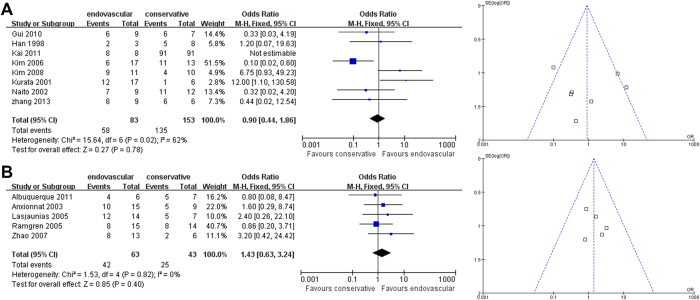
**Analysis of Overall Good Recovery Outcomes.** Forest plots and funnel plots of (**A**) East Asian and (**B**) non-East Asian studies.

Third, the pooled outcomes for good recovery for endovascular therapy versus non-interventional therapy were separately compared in East Asian and non-East Asian studies. Both East Asians and non-East Asians showed no differences in treatment effect between endovascular therapy versus non-interventional therapy on good recovery outcomes (East Asian OR [95% CI]: 0.90 [0.44-1.86], *P* = 0.78, Fig. 6A; non-East Asian OR [95% CI]: 1.43 [0.63-3.24], *P* = 0.40, Fig. 6B). For the East Asian comparison, there was significant heterogeneity (I^2^ = 62%, Fig. 6A). Sensitivity analysis to investigate possible sources of heterogeneity in the included studies indicated that no single study was an important source of heterogeneity; that is, exclusion of no individual study from the overall meta-analysis significantly changed the *p*-value of heterogeneity. For the non-East Asian comparison (Fig. 6B), sensitivity analysis revealed that the summary effect estimates and 95% CI significantly changed (*p* < 0.05), indicating that this particular finding was not particular robust. For the East Asian comparison, Begg’s test (*P* = 0.386) and Egger’s test (*P* = 0.203) revealed no significant publication bias. For the non-East Asian comparison, Begg’s test (*P* = 0.462) and Egger’s test (*P* = 0.314) revealed no significant publication bias.

**Figure 7:**
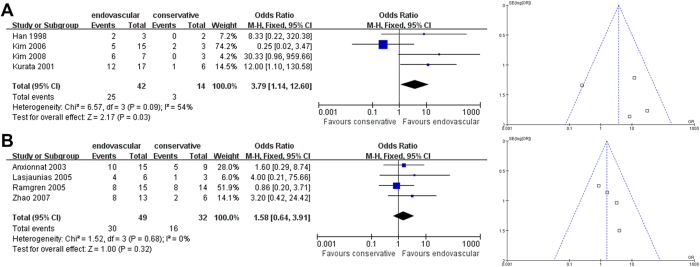
**Analysis of Subgroup Good Recovery Outcomes for Patients with Ruptured Dissections.** Forest plots and funnel plots of (**A**) East Asian and (**B**) non-East Asian studies.

However, the subgroup good recovery analysis for CCAD patients with ruptured dissections revealed that endovascular therapy is significantly superior to non-interventional therapy for East Asians (East Asian OR [95% CI]: 3.79 [1.14-12.60], *P* = 0.03; Fig. 7A) with medium heterogeneity between the included studies (I^2^ = 54%). Sensitivity analysis to investigate possible sources of heterogeneity in the included studies indicated that no single study was an important source of heterogeneity; that is, exclusion of no individual study from the overall meta-analysis significantly changed the *p*-value of heterogeneity. No differences in treatment effect on good recovery outcomes were observed between the two approaches for non-East Asian CCAD patients with ruptured dissections (non-East Asian OR [95% CI]: 1.58 [0.64-3.91], *P* = 0.32; Fig. 7B). For the non-East Asian comparison (Fig. 7B), sensitivity analysis revealed that the summary effect estimates and 95% CI significantly changed (*p* < 0.05), indicating that this particular finding was not particular robust. For the East Asian comparison, Begg’s test (*P* = 1.000) revealed no significant publication bias (Egger’s test was not performable). For the non-East Asian comparison, Begg’s test (*P* = 0.308) and Egger’s test (*P* = 0.106) revealed no significant publication bias.

## Disscussion

The aim of this systematic review and meta-analysis will be to compare the clinical outcomes of endovascular versus non-interventional therapy for CCAD in East Asian and non-East Asian populations. We found that endovascular therapy is significantly superior to non-interventional therapy for East Asian CCAD patients with ruptured dissections in terms of mortality and good recovery outcomes. That being said, we found no differences in treatment effect upon mortality, disability, or good recovery outcomes between endovascular therapy and non-interventional therapy for the CCAD populations-at-large nor for non-East Asian CCAD patients with ruptured dissections.

The current findings slightly conflict with a previous meta-analysis by Chen *et al.*, which showed that endovascularly-treated CCAD patients showed a significantly lower mortality than non-interventional CCAD patients^5^. Chen *et al.* noted that this significant outcome was concealed when the East Asian study by Kurata *et al.* or Jin *et al.* was omitted^5^,^19^,^23^. This sensitivity analysis by Chen *et al.* revealed that these two East Asian studies were driving the mortality findings for the meta-analysis as a whole. Here, by purposefully separating the East Asian and non-East Asian studies, we were able to demonstrate no significant differences in mortality outcomes in either population-at-large.

Moreover, in Chen *et al.*’s ruptured dissection subgroup analysis, endovascular treatment was associated with reduced mortality and a higher rate of good recovery but no significant difference in disability rate in CCAD patients with ruptured dissections. Here, we found that the reduced mortality and higher rate of good recovery only applies to East Asian CCAD patients with ruptured dissections, not to non-East Asian CCAD patients. These findings exemplify the importance of ethnicity-based subgroup analyses for interventional meta-analysis, as drug therapies can have differential effects upon various ethnic populations due to genetic diversity^31^.

In terms of interpretation, there are at least two reasons that may explain the observed superiority of endovascular treatment over non-interventional therapy in East Asian CCAD patients with ruptured dissections. First. previous studies have reported that the risk of critical bleeding may be especially higher among East Asian patients undergoing anti-thrombotic therapy^7^. For example, warfarin-related intracranial hemorrhage in East Asian patients was reported to be 1.75 per 100 patient-years, which is significantly higher than the figure in Caucasians of 0.34 per 100 patient-years^7^,^32^. This increased risk of critical bleeding associated with anti-thrombotic therapy in East Asians may explain the observed superiority of endovascular treatment over non-interventional therapy in East Asian CCAD patients with ruptured dissections. Second, differential prescribing behaviors by health care providers in East Asia and the West may be partly responsible for the observed findings. For example, Chinese and Japanese clinicians have been shown to underprescribe warfarin in favor of anti-platelet therapies such as aspirin in atrial fibrillation patients (which is against the recommended course of action in such patients)^7^,^33^,^34^. Such prescribing behaviors may adversely affect the efficacy of non-interventional care of East Asian CCAD patients with ruptured dissections, thereby making endovascular treatment appear superior by comparison.

Sensitivity analysis was used to investigate possible sources of heterogeneity in the comparisons with significant heterogeneity (I^2^ > 50%); namely, the East Asian mortality analysis, East Asian good recovery analysis, and East Asian good recovery subgroup analysis for ruptured dissections (Figs. 2A,6A, and 7A). All indicated that no single study was an important source of heterogeneity. On this basis, the source of heterogeneity is multi-factorial and is likely related to a combination of patient factors (e.g., age, gender, ethnicity, body mass index, and disease status), operator factors (individual experience and learning curves for each device), procedural factors (e.g., puncture site, sheath size, first versus repeat procedure, level of anticoagulation (if any), and adjunctive pharmacotherapy (if any), health system factors (e.g., differing standards of medical care across study institutions, differing health service quality levels), and varying follow-up durations.

Moreover, sensitivity analysis was performed by iteratively removing one study at a time to confirm that our findings were not driven by any single study. We found that the summary effect estimates and 95% CI significantly changed for the non-East Asian disability analysis, non-East Asian good recovery analysis, and non-East Asian good recovery subgroup analysis for ruptured dissections (Figs. 4B,6B, and 7B), indicating that these particular findings are not particular robust. Fortunately, this finding does not affect our main conclusions as these particular comparisons were all non-significant.

There are several limitations to this study. First, this meta-analysis was unable to analyze the underlying covariate factors (e.g., smoking status, hypertension and obesity) that may have influenced the observed differences between East Asians versus non-East Asians^35^. Thus, future studies assessing outcomes in CCAD patients should specifically report covariate data on their participants by ethnic group in order to enable meta-analysis of these factors. Second, aside from the differential prescribing behaviors alluded to above, there may be systemic differences in endovascular operator training and skill, endovascular device quality, and post-intervention medical management between the East Asian and non-East Asian study sites that may have contributed to the observed differences^36^. Third, the categorization of ‘East Asian’ versus ‘non-East Asian’ was empirically based on the location of the study site. Since most ‘East Asian’ study sites are very homogenous in terms of ethnicity (Harvard Institute of Economic Research (HIER) ethnic fractionalization indices for China, Japan, and South Korea: 0.1538, 0.0119, and 0.0020, respectively), the same cannot be said for the included American study (e.g., HIER ethnic fractionalization index for the USA: 0.4901)^37^. Thus, the ethnic heterogeneity of the included American study may have adversely affected the meta-analysis; thus, future studies assessing CCAD outcomes should segregate patients into ethnic subgroups in order to enable race-specific data reporting. Fourth, a selective reporting bias may exist as several studies failed to report all outcomes^38^. Fifth, we were unable to determine the precise factors responsible for the significant heterogeneity observed in the East Asian mortality analysis, East Asian good recovery analysis, and East Asian good recovery subgroup analysis for ruptured dissections. Sixth, as in any meta-analysis, publication bias is a potential limitation to interpretation; Egger’s and Begg’s testing revealed publication bias for the non-East Asian mortality analyses (Figs. 2B,3B). Therefore, these findings should be interpreted with caution.

In conclusion, endovascular therapy appears to be superior to non-interventional therapy for East Asian CCAD patients with ruptured dissections in terms of mortality and good recovery outcomes. Based on this evidence, endovascular therapy should be especially advisable in East Asian CCAD patients with ruptured dissections. However, this study provides no evidence to preferentially support endovascular therapy over non-interventional therapy in non-East Asian CCAD patients in terms of mortality, disability, and good recovery outcomes.

## Author Contributions

Conceived and designed the study: L.H.Y., Y.S. and R.Z.H. Performed the experiments: R.Z.H., Y.L.W. and Y.W. Analyzed the data: L.C.N., G.W., W.J. and L.J. Drafted the manuscript: Y.S. and R.Z.H.

## Additional Information

**How to cite this article**: Huang, R. *et al.* Endovascular versus Non-Interventional Therapy for Cervicocranial Artery Dissection in East Asian and Non-East Asian Patients: a Systematic Review and Meta-analysis. *Sci. Rep.* **5**, 10474; doi: 10.1038/srep10474 (2015).

## Figures and Tables

**Table 1:** Characteristics of Included Studies.

**Study**	**Country**	**Design**	**Participants (n)**	**Male (%)**	**Mean age (yrs)**	**Mean follow-up (mths)**	**PCD (%)**	**Ruptured dissection (%)**	**DA (%)**	**Method for evaluating functional outcome and end points**
										
***East Asian Studies (n=11)***										
Chung 2002	Korea	Retro	23	NA	NA	53	NA	12/23	NA	Death, residual deficit, resolved or improved, excellent
Deng 2011	China	Retro	21	17/21	50.1	12.1	21/21	NA	21/21	GOS; death, VS, SD, MD, good recovery, re-bleeding
Gui 2010	China	Pro	16	13/16	39.2	NA	16/16	1/16	7/16	mRS
Han 1998	Korea	Retro	11	11/11	39.1	60	11/11	5/11	5/11	Death, hemiparesis and dysphasia, re-bleeding, recurrent ischemia, excellent
Jin 2013	China	Retro	71	53/71	51.1	12	NA	NA	NA	mRS; death, favorable outcome (mRS score>4), poor outcome (mRS score≤3)
Kai 2011	Japan	Retro	99	NA	NA	24	99/99	0/99	99/99	mRS
Kim 2006	Korea	Retro	30	25/30	43.8	19.2	30/30	18/30	15/30	mRS; death, poor (mRS score, 4-5), moderate (mRS score, 2-3), good (mRS score, 0-1)
Kim 2008	Korea	Retro	21	12/21	53	21.5	21/21	10/23	9/23	mRS, death, poor outcome (mRS score, 4-5), favorable outcome (mRS score, 0-2), re-bleeding, recurrent ischemia
Kurata 2001	Japan	Retro	23	18/23	54.5	9	23/23	23/23	23/23	GOS, death, VS, SD, MD, good recovery, re-bleeding
Naito 2002	Japan	Retro	21	13/21	49.7	14	21/21	3/21	14/21	GOS; death, VS, SD, MD, good recovery
Zhang 2013	China	Retro	15	9/15	44	6	15/19	0/15	7/15	recurrent ischemia
										
***Non-East Asian Studies (n=5)***										
Albuquerque 2011	USA	Pro	13	5/13	44	19	10/13	0/13	NA	Death, permanent neurologic deficit, good recovery
Anxionnat 2003	France	Retro	24	12/24	49.5	NA	23/24	24/24	23/24	GOS, death, VS, SD, MD, good recovery, re-bleeding
Lasjaunias 2005	France	Retro	21	12/21	NA	NA	11/21	9/21	21/21	Death, stable, survived, cured, lost to follow-up
Ramgren 2005	Sweden	Retro	29	18/25	55	6	29/29	29/29	20/23	GOS; death, VS, SD, MD, good recovery, re-bleeding, recurrent ischemia
Zhao 2007	France	Retro	19	11/19	44.5	NA	19/19	19/19	15/19	Karnovsky score

*GOS scoring: 5 = good recovery, 4 = moderate disability, 3 = severe disability, 2 = vegetable state, and 1 = death. Abbreviations: DA, dissecting aneurysm; GOS, Glasgow Outcome Scale; MD, moderate disability; mRS, modified Rankin Scale; NA, not available; pro, prospective study; PCD, posterior circulation dissection; retro, retrospective study; SD, severe disability; VS, vegetative state.

**Table 2:** Quality Assessment of Included Studies.

**Study**	**Selection**			**Comparability**			**Outcome Measures**			**Total**
	**Recruitment criteria reported?**	**Representativeness of participants to the general patient population?**	**Both treatment groups drawn from the same population?**	**Outcomes of interest not present at study start?**	**Comparability of groups in terms of age, Hunt/Hess grade, dissection location?**	**Control for potential confounders?(by matching, modeling, etc.)**	**Pre-specification of outcomes?**	**Adequacy of follow-up length?**	**Adequacy of follow-up %?**	
										
***East Asian Studies (n=11)***										
Chung 2002	1	1	1	1	1	0	1	1	1	**8**
Deng 2011	1	0	1	1	1	0	1	1	1	**7**
Gui 2010	1	1	1	1	1	0	1	1	1	**8**
Han 1998	1	1	1	1	1	0	1	1	1	**8**
Jin 2013	1	1	1	1	1	0	1	1	1	**8**
Kai 2011	1	1	1	1	1	0	1	1	1	**8**
Kim 2006	1	1	1	1	1	0	1	1	1	**8**
Kim 2008	1	1	1	1	1	0	1	1	1	**8**
Kurata 2001	1	1	1	1	1	0	1	1	1	**8**
Naito 2002	1	1	1	1	1	0	1	1	1	**8**
Zhang 2013	1	1	1	1	1	0	1	1	1	**8**
										
***Non-East Asian Studies (n=5)***										
Albuquerque 2011	1	0	1	1	1	0	1	1	1	**7**
Anxionnat 2003	1	0	1	1	1	0	1	1	1	**7**
Lasjaunias 2005	1	0	1	1	1	0	1	1	1	**7**
Ramgren 2005	1	1	1	1	1	0	1	1	1	**8**
Zhao 2007	1	1	1	1	1	0	1	1	1	**8**
